# Phase I study of liposomal irinotecan in patients with metastatic breast cancer: findings from the expansion phase

**DOI:** 10.1007/s10549-020-05995-7

**Published:** 2020-11-17

**Authors:** Jasgit C. Sachdev, Pamela Munster, Donald W. Northfelt, Hyo Sook Han, Cynthia Ma, Fiona Maxwell, Tiffany Wang, Bruce Belanger, Bin Zhang, Yan Moore, Arunthathi Thiagalingam, Carey Anders

**Affiliations:** 1 HonorHealth Research Institute, 10510 N. 92nd Street, Suite 200, Scottsdale, AZ 85258 USA; 2 Translational Genomics Research Institute, Phoenix, AZ USA; 3 University of California, San Francisco, CA USA; 4 Mayo Clinic Hospital, Phoenix, AZ USA; 5 Moffitt Cancer Center, Tampa, FL USA; 6 Washington University, St. Louis, MO USA; 7 Ipsen, Abingdon, UK; 8 Ipsen, Cambridge, MA USA; 9 Duke Cancer Institute, Durham, NC USA

**Keywords:** Liposomal irinotecan, Metastatic breast cancer, Objective response rate, Phase I clinical trial, Brain metastases, Heavily pretreated patients

## Abstract

**Purpose:**

Metastatic breast cancer (mBC) remains incurable and is associated with low survival rates. This study assessed the efficacy and safety of liposomal irinotecan in heavily pretreated patients with mBC, with or without active brain metastases (BM).

**Methods:**

Following the dose escalation phase and determination of recommended phase 2 dose, the expansion phase of this phase I, open-label, non-randomized study, assigned adult women to cohorts based on mBC subtype: cohort 1, hormone receptor +/human epidermal growth factor receptor 2−; cohort 2, triple-negative breast cancer; or cohort 3, any mBC subtype with active BM. Patients received liposomal irinotecan 50 or 70 mg/m^2^ free base every 2 weeks. Here, we report secondary outcomes including best overall response (BOR), objective response rate (ORR), and treatment-emergent adverse events (TEAEs).

**Results:**

For non-central nervous system (non-CNS) disease across all cohorts (intent-to-treat population, *N* = 29), the ORR was 34.5% (95% confidence interval: 17.94–54.33), with a BOR of partial response in 10 patients (34.5%), stable disease in five (17.2%), progressive disease in 10 (34.5%); four patients were unevaluable (13.8%). The ORR for the CNS cohort was 30.0% (95% confidence interval: 6.67–65.25) using modified Response Evaluation Criteria in Solid Tumors. Common grade 3 or higher TEAEs were diarrhea (27.6%), nausea (17.2%), fatigue (13.8%), asthenia (10.3%), and hypokalemia (10.3%). Serious treatment-related TEAEs were reported in six patients (20.7%). No treatment-related TEAEs resulted in death.

**Conclusions:**

Liposomal irinotecan monotherapy demonstrated antitumor activity in heavily pretreated patients with mBC, with or without BM. The observed safety profile was consistent with that in previous studies.

**Clinical trial registration:** Trial registration ID NCT01770353.

**Electronic supplementary material:**

The online version of this article (10.1007/s10549-020-05995-7) contains supplementary material, which is available to authorized users.

## Introduction

An estimated 276,480 women in the USA are predicted to be diagnosed with invasive breast cancer in 2020 [1]. Despite recently improved outcomes, the 5-year survival rate for women with metastatic breast cancer (mBC) in the USA remains low at 27% [1]. The estimated incidence of brain metastases (BM) in patients with mBC is 24%; the estimated survival time of patients with BM is 15 months from diagnosis. BM are associated with morbidity and negatively impact functional status and quality of life [2]. Because women with mBC are living longer, cases of BM are expected to increase.

Systemic treatments for mBC are selected based on multiple factors, including age, comorbidities, hormone receptor and human epidermal growth factor receptor 2 (HER2) status, previous cancer treatments, and tumor burden [3]. In patients with mBC and BM, systemic treatments have limited central nervous system (CNS) efficacy, and disease progression after localized treatment(s) (i.e., whole brain radiation therapy, stereotactic radiosurgery, and/or surgical resection) presents a significant clinical challenge [2]. Currently, tucatinib, in combination with trastuzumab and capecitabine, is the only systemic therapy approved by the Food and Drug Administration (FDA) for the treatment of adult patients with advanced unresectable or metastatic HER2+ breast cancer, including patients with BM, who have received one or more prior anti-HER2-based regimens in the metastatic setting [4].

The anticancer prodrug irinotecan has a mechanism of action that is distinct from other medications used for mBC treatment; therefore, the risk of cross-resistance from previous cancer therapies is considered low. The active metabolite of irinotecan, SN-38, reversibly binds to the topoisomerase I–DNA complex and prevents religation of single-strand breaks, leading to double-strand DNA damage and cell death [5]. Irinotecan can cross the blood–brain barrier and has shown promising results for BM treatment in two phase II studies; one in primary CNS tumors [6] and another in triple-negative breast cancer (TNBC) when administered with iniparib [7].

Liposomal irinotecan (ONIVYDE®; Ipsen Biopharmaceuticals, Inc.; historical names include nal-IRI, MM-398, or PEP02) is an intravenously delivered formulation [5]; individual liposomes have a diameter of approximately 110 nm [8], which is close to the nanoscale (1–100 nm) [9]. Liposomal encapsulation increases the nominal plasma half-life of irinotecan [10]. Deposition in tumor lesions occurs through leaky vasculature within the lesion via the enhanced permeability and retention (EPR) [11] effect before conversion of the payload to SN-38 [12]. Liposomal irinotecan is approved, in combination with 5-fluorouracil and leucovorin, for the treatment of patients with metastatic pancreatic ductal adenocarcinoma (mPDAC) following progression with gemcitabine-based therapy [5]. Preclinical data suggest that liposomal irinotecan may have utility as a treatment for mBC with BM [13].

To investigate this potential activity in heavily pretreated patients with mBC, including active BM, we report efficacy and safety outcomes from the expansion phase of a phase I cross-indication translational study (ClinicalTrials.gov identifier: NCT01770353).

## Methods

### Study design

**Fig. 1:**
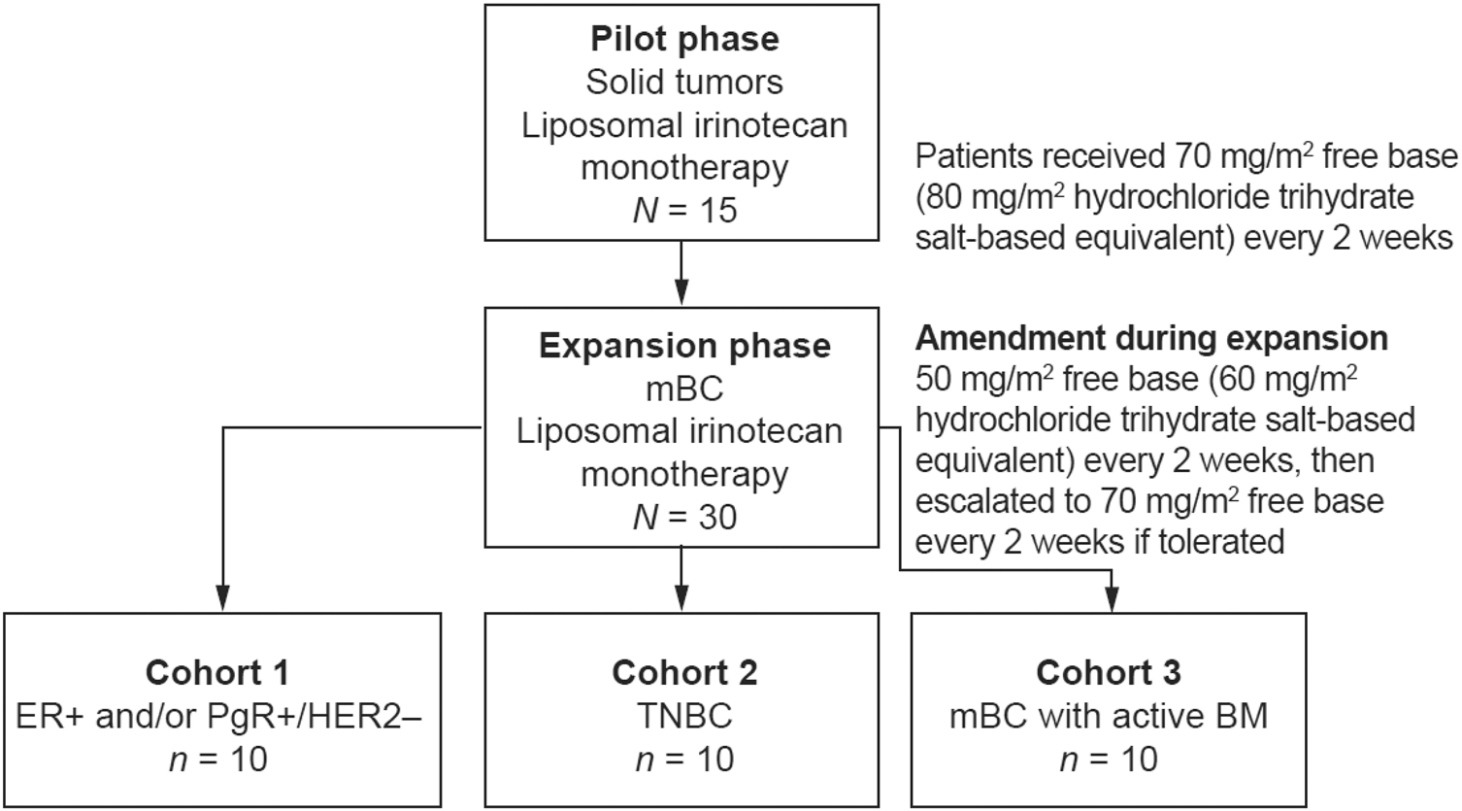
Study design. *BM* brain metastases, *ER* estrogen receptor, *HER2* human epidermal growth factor receptor 2, *mBC* metastatic breast cancer, *PgR* progesterone receptor, *TNBC* triple-negative breast cancer

This multicenter, non-comparative, open-label, non-randomized, phase I study was conducted in the USA between November 19, 2012 (first patient, first visit) and October 2, 2018 (last patient, last visit). The study comprised a pilot phase followed by an expansion phase (Fig. 1). The pilot phase was previously reported by Ramanathan et al. [14]. The expansion phase was conducted in patients with mBC (first liposomal irinotecan dose in first patient, May 18, 2015), and the primary outcome was to investigate ferumoxytol quantitation in tumor lesions (to be reported elsewhere).

Here, we report secondary outcomes from the expansion phase: the efficacy and safety of liposomal irinotecan in adult patients with mBC, including active BM.

### Expansion phase population

Adult women aged ≥ 18 years were recruited into three cohorts (target 10 patients per cohort) based on historical archival receptor subtyping: cohort 1, estrogen receptor (ER)+ and/or progesterone receptor (PgR)+/HER2−; cohort 2, ER− and PgR−/HER2− (TNBC); or cohort 3, any mBC subtype with active BM (mBCBM). In cohort 3, patients were required to have radiographic evidence of new or progressive BM after radiation therapy with ≥ 1 lesion measuring ≥ 1 cm in the longest dimension on gadolinium-enhanced MRI, and to be considered neurologically stable. In all cohorts, key inclusion criteria included locally advanced or metastatic disease with ≥ 2 radiologically measurable lesions; an Eastern Cooperative Oncology Group performance status of 0 or 1; adequate bone marrow reserves, and adequate hepatic and renal function; and 1–5 prior lines of chemotherapy in the metastatic setting. Patients with active CNS metastases were excluded from cohorts 1 and 2. See Supplementary Table S1 for full inclusion and exclusion criteria, including those specifically relating to patients with BM.

### Expansion phase treatment

On day 1, patients received a single dose of intravenous ferumoxytol 5 mg/kg infused over 15 min, used here as a magnetic resonance imaging (MRI) lesion-imaging agent (reported elsewhere) [14, 15]. Within 7 days after ferumoxytol infusion, patients received their first dose of intravenous liposomal irinotecan 70 mg/m^2^ free base, infused over 90 min. Extracranial biopsies from a single lesion were acquired either prior to dosing with liposomal irinotecan or approximately 72 h after the first dose. Subsequent doses of liposomal irinotecan were administered every 2 weeks (± 2 days). Protocol Amendment 4 (November 3, 2016) reduced the starting dose to 50 mg/m^2^ free base, with allowance for escalation to 70 mg/m^2^ free base, depending on patient tolerance.

In all cohorts, treatment continued until disease progression was observed (as assessed using Response Evaluation Criteria in Solid Tumors [RECIST] v1.1 criteria), unacceptable tolerability had occurred, or consent was withdrawn. In cohorts 1 and 2, treatment could continue in patients with radiographic disease progression without symptomatic deterioration if they had derived clinical benefit, based on a consensus between the investigator, medical monitor, and sponsor. In cohort 3, CNS and non-CNS disease were assessed separately, and treatment was discontinued in patients with radiographic evidence of CNS disease progression. Treatment continuation was permitted, at the investigator’s discretion, in patients with non-CNS disease progression in the absence of CNS disease progression. Cohort 3 patients with symptomatic CNS disease progression but without radiographic confirmation were permitted to continue treatment.

During the study, all concurrent medical conditions and complications of the underlying malignancy could be treated at the discretion of the investigator, according to acceptable local standards of medical care. Patients could receive analgesics, antiemetics, antibiotics, anti-pyretics, and blood products as deemed necessary. Further details of concomitant therapy are provided in supplementary materials. Information on post-trial treatments was not collected.

### Efficacy assessments

Efficacy assessments were included in the expansion phase as secondary study endpoints; primary endpoints did not include efficacy and will be reported elsewhere. Tumor assessments, both non-CNS (all cohorts) and CNS (cohort 3 only) by computed tomography (CT) or magnetic resonance imaging, were performed at baseline and at 8-week intervals. RECIST v1.1 and modified RECIST (Supplementary Table S2) were utilized to assess non-CNS systemic disease and CNS disease, respectively. To be considered evaluable, patients were required to have received liposomal irinotecan and to have completed at least one CT scan at the 8-week post-treatment time point. For post-baseline assessments, overall tumor response was classified as complete response (CR), partial response (PR), stable disease (SD), progressive disease (PD), or not evaluable (NE). A non-CR/non-PD was also available for non-target lesions. Based on tumor response assessments, additional efficacy outcomes included best overall response (BOR), objective response rate (ORR), clinical benefit rate (CBR; defined as CR or PR, and SD lasting at least 24 weeks), duration of objective response (DOR), and progression-free survival (PFS); definitions are provided in supplementary materials.

### Safety assessments

All treatment-emergent adverse events (TEAEs) were recorded using National Cancer Institute Common Terminology Criteria for Adverse Events (NCI CTCAE) version 4.02. Final safety assessments were completed 30 days (± 7 days) after the patient received their last dose of liposomal irinotecan.

### Metastatic tumor receptor status—exploratory, post hoc analyses

See Supplementary Materials for details of retrospective analyses of metastatic tumor receptor status for on-study biopsy materials.

### Statistical analysis

All patients who received at least one dose of liposomal irinotecan were included in the liposomal irinotecan safety population. The liposomal irinotecan efficacy population comprised all patients who received liposomal irinotecan and had evaluable efficacy data. No formal hypothesis testing was performed; therefore, this study was not powered to detect statistical differences in any parameter. Descriptive results are reported.

## Results

### Patients

A patient disposition flowchart is provided in Supplementary Fig. S1. In total, 30 patients (10 per cohort) were enrolled in the expansion phase (Table 1). All enrolled patients were women with a median age of 53 (range 29–70) years. In each cohort, most patients were white, and most were heavily pretreated with a median of three (range 0–6) prior cytotoxic anticancer regimens in the metastatic setting.

**Table 1:** Demographics and baseline characteristics (ferumoxytol safety population, *N* = 30)

	Cohort 1 (*n* = 10)	Cohort 2 (*n* = 10)	Cohort 3^a^ (*n* = 10)	Total population (*N* = 30)
Sex, female, *n* (%)	10 (100)	10 (100)	10 (100)	30 (100)
Age, years, median (range)	56.0 (49–68)	52.5 (37–70)	45.5 (29–63)	53.0 (29–70)
Race, *n* (%)				
White	8 (80.0)	8 (80.0)	7 (70.0)	23 (76.7)
Black or African American	0	1 (10.0)	1 (10.0)	2 (6.7)
American Indian or Native Alaskan	0	0	0	0
Asian	1 (10.0)	0	1 (10.0)	2 (6.7)
Native Hawaiian or other Pacific islander	1 (10.0)	0	0	1 (3.3)
Other	0	1 (10.0)	1 (10.0)	2 (6.7)
Time since metastatic diagnosis, months, median (range)	63.7 (16–87)	20.7 (0–34)	32.4 (8–55)	24.0 (0–87)
Number of prior cytotoxic anticancer regimens, median (range)	3.0 (1–6)	3.0 (0–5)	3.0 (1–6)	3.0 (0–6)

Percentages are subject to rounding

*BM* brain metastases, *ER* estrogen receptor, *HER2* human epidermal growth factor receptor 2, *mBC* metastatic breast cancer, *PgR* progesterone receptor, *TNBC* triple-negative breast cancer

^a^Four patients had TNBC; three patients were ER+ and/or PgR+/HER2+, two patients were ER+ and/or PgR+/HER2−, and one patient was ER− and/or PgR−/HER2+. Of the four patients with HER2+ mBC based on pre-study biopsies, three had received HER2 blockade prior to study entry. The patient who had not received prior HER2 blockade had TNBC before their diagnosis of BM. Brain tissue biopsy from this patient showed ER+ HER2+ tissue. At diagnosis of BM, after surgery, radiotherapy and letrozole, the patient had stable BM and was subsequently enrolled in the expansion phase of the present clinical trial

The safety population comprised 29 patients who had received at least one dose of liposomal irinotecan. All these patients had evaluable efficacy data and comprised the liposomal irinotecan efficacy population. One patient enrolled in cohort 2 (TNBC) died owing to PD before receiving liposomal irinotecan and was not included in the liposomal irinotecan safety or efficacy analyses.

**Fig. 2:**
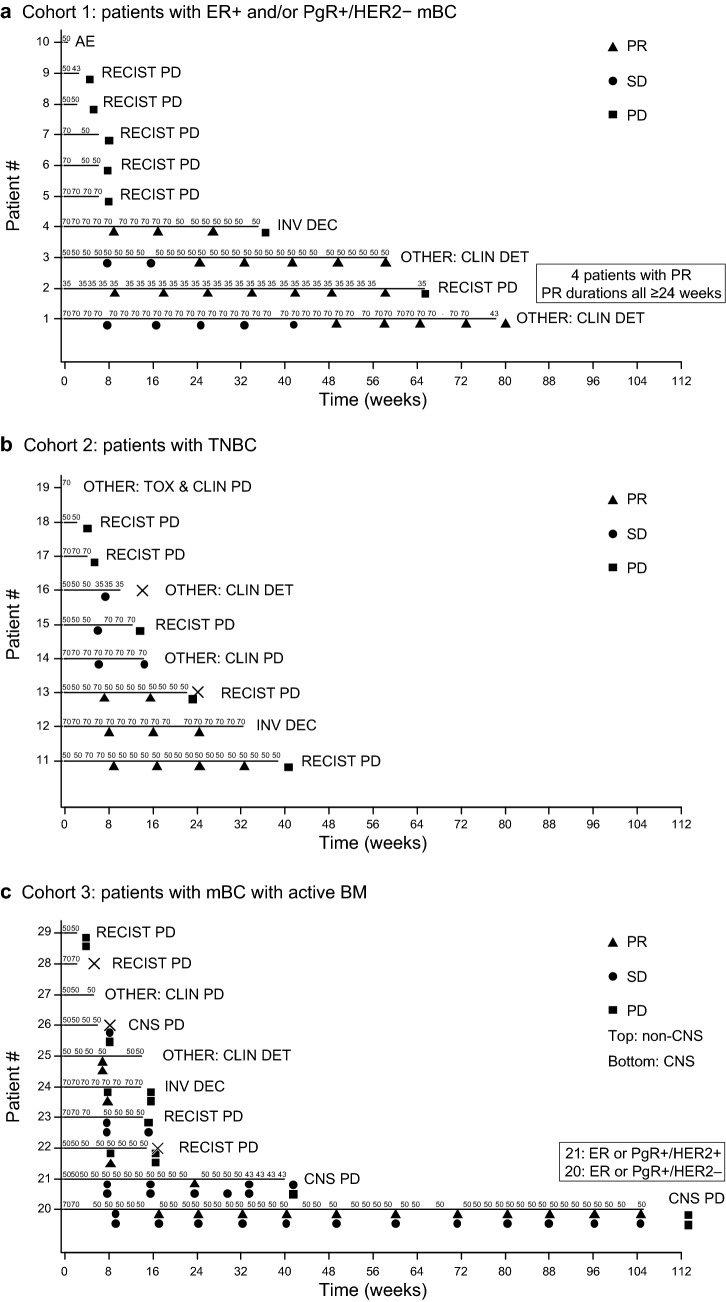
Response to liposomal irinotecan over time until treatment discontinuation (liposomal irinotecan efficacy population, *n* = 29). Individual doses of liposomal irinotecan are displayed above each patient’s treatment timeline, and the values depict a 35, 43, 50, or 70 mg/m^2^ free base intravenous infusion. One patient in cohort 2 was enrolled but discontinued before receiving treatment. Patient death is depicted by an ‘X’. *AE* adverse event, *BM* brain metastases, *CLIN* clinical, *CNS* central nervous system, *DET* deterioration, *ER* estrogen receptor, *HER2* human epidermal growth factor receptor 2, *INV DEC* investigator decision, *mBC* metastatic breast cancer, *PD* progressive disease, *PgR* progesterone receptor, *PR* partial response, *RECIST* Response Evaluation Criteria in Solid Tumors, *SD* stable disease, *TNBC* triple-negative breast cancer, *TOX* toxicity

In total, 13 patients initiated liposomal irinotecan at 70 mg/m^2^ free base, and 15 patients initiated at 50 mg/m^2^ free base. One patient initiated liposomal irinotecan at 35 mg/m^2^ free base based on the investigator’s clinical decision and their UGT1A1*28 allele homozygous polymorphism. Median exposure to liposomal irinotecan among all patients was 12.3 (range 0.1–105.3) weeks. All patients had discontinued the study by week 114 (Fig. 2). Reasons for discontinuation of liposomal irinotecan were PD radiographically confirmed as per RECIST v1.1 (18 patients, 62.1% [percentages rounded]), other (seven patients, 24.1%), investigator decision (three patients, 10.3%), and TEAEs (one patient, 3.4%). ‘Other’ was an option on the electronic case report form and included clinical deterioration or clinical PD in six patients, and toxicity-related diarrhea and clinical PD in one patient.

### Efficacy

#### Response to treatment

The BOR for non-CNS disease across all cohorts was PR in ten patients (34.5%), SD in five patients (17.2%), and PD in ten patients (34.5%); four patients (13.8%) were NE (Table 2). No patients had a CR. The BOR was: cohort 1 (ER+ and/or PgR+/HER2−), PR in 40.0%, and PD in 50.0%, with one patient NE; cohort 2 (TNBC), PR in 33.3%, SD in 33.3%, and PD in 22.2%, with one patient NE; cohort 3 (mBCBM) for non-CNS disease, PR in 30.0%, SD in 20.0%, and PD in 30.0%, with two patients NE. For non-CNS disease across all cohorts, both the ORR and the CBR were 34.5% (Table 2). For CNS disease in cohort 3, the BOR was PR in 30.0%, SD in 30.0%, and PD in 20.0%, with two patients NE, and the ORR and CBR were 30.0% and 50.0%, respectively (Table 2).

**Table 2:** Response to treatment (evaluable efficacy population, *N* = 29)

	Cohort 1 (*n* = 10)	Cohort 2^a^ (*n* = 9)	Cohort 3		Total population (non-CNS)
			non-CNS (*n* = 10)	CNS (*n* = 10)	(*N* = 29)
Best overall response, *n* (%)					
Complete response	0	0	0	0	0
Partial response	4 (40.0)	3 (33.3)	3 (30.0)	3 (30.0)	10 (34.5)
Stable disease	0	3 (33.3)	2 (20.0)	3 (30.0)	5 (17.2)
Progressive disease	5 (50.0)	2 (22.2)	3 (30.0)	2 (20.0)	10 (34.5)
Not evaluable	1 (10.0)	1 (11.1)	2 (20.0)	2 (20.0)	4 (13.8)
Objective response rate					
Patients with a complete or partial response, *n* (%)	4 (40.0)	3 (33.3)	3 (30.0)	3 (30.0)	10 (34.5)
95% CI	12.16–73.76	7.49–70.07	6.67–65.25	6.67–65.25	17.94–54.33
Clinical benefit rate					
Patients with a complete or partial response, or stable disease that lasted at least 24 weeks, *n* (%)	4 (40.0)	3 (33.3)	3 (30.0)	5 (50.0)	10 (34.5)
95% CI	12.16–73.76	7.49–70.07	6.67–65.25	18.71–81.29	17.94–54.33
Duration of objective response					
Number of months, median (range)	7.46 (6.4–13.0)	5.62 (3.7–7.4)	4.14 (0.0–22.2)	1.84 (0.0–1.9)	6.74 (0.0–22.2)
Progression-free survival					
Number of months, median (range)	1.9 (1.1–15.1)	4.3 (1.0–9.4)	3.2 (0.9–26.1)	3.6 (0.9–9.6)	3.2 (1.8–8.4)

CNS tumor response was evaluated according to modified RECIST

Percentages are subject to rounding

*CI* confidence interval, *CNS* central nervous system, *RECIST* Response Evaluation Criteria in Solid Tumors

^a^One patient was enrolled but not treated with liposomal irinotecan and is not included in any safety or efficacy assessments. Non-CNS tumor response was evaluated according to RECIST v1.1

#### Duration of response

In cohort 1 (ER+ and/or PgR+/HER2−), PR was reported in four patients; it lasted at least 24 weeks in three of the patients and more than 48 weeks in the fourth patient (Fig. 2a). In cohort 2 (TNBC), PR was reported in three patients; it lasted approximately 24 weeks in two of the patients and 40 weeks in the third patient (Fig. 2b). In cohort 3 (mBCBM), non-CNS PR was reported in three patients; CNS PR was reported in three patients (all of whom had TNBC) at week 8, but not at a subsequent assessment (Fig. 2c). One patient (TNBC) in cohort 3 had a non-CNS and a CNS PR at week 8, and another patient (ER+ and/or PgR+/HER2−) had a non-CNS PR from week 16 to 114 and a best CNS response of SD over the same time period).

Across all cohorts for non-CNS disease, median (range) DOR and PFS were 6.74 (0.0–22.2) months and 3.2 (1.8–8.4) months, respectively (Table 2). For CNS disease in cohort 3 (mBCBM), median (range) DOR and PFS were 1.84 (0.0–1.9) months and 3.6 (0.9–9.6) months, respectively (Table 2).

#### Tumor lesion size

**Fig. 3:**
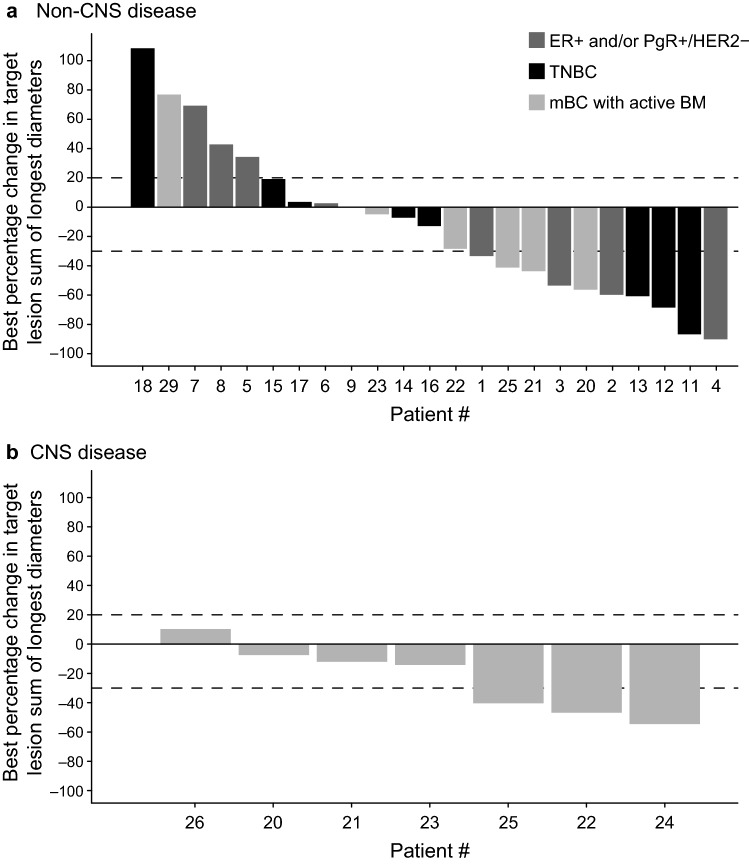
Percentage reduction in tumor lesion size in patients with (**a**) non-CNS disease and (**b**) CNS disease^a^. ^a^Only patients who had an evaluable post-baseline tumor assessment for designated target lesions are included in the waterfall plots. Horizontal dashed lines represent the range of stable disease (lower line, < 30% reduction in tumor size; upper line, < 20% increase in tumor size), as per RECIST v1.1 criteria. Patient numbers on the x axis relate to patient numbers on Fig. 2. *BM* brain metastases, *CNS* central nervous system, *ER* estrogen receptor, *HER2* human epidermal growth factor receptor 2, *mBC* metastatic breast cancer, *PgR* progesterone receptor, *RECIST* Response Evaluation Criteria in Solid Tumors, *TNBC* triple-negative breast cancer

There were 23 patients across all cohorts with measurable non-CNS disease with evaluable follow-up; seven patients in cohort 3 had measurable CNS disease and evaluable follow-up (Fig. 3). A reduction in tumor lesion size was observed in the majority of patients for both non-CNS disease (14 of 23 patients, 60.9%; Fig. 3a) and CNS disease (six of seven patients, 85.7%; Fig. 3b). In cohort 2 (TNBC) and for the non-CNS disease in cohort 3 (mBCBM), a reduction was observed in more than half of the evaluable patients. Compared with baseline measures, reductions varied from 5 to 90% for non-CNS lesions and from 7 to 55% for CNS lesions.

### Safety

Median duration of exposure to liposomal irinotecan across all cohorts was 12.3 (0.1–105.3) weeks (Table 3). A dose reduction was recorded in six patients who initiated liposomal irinotecan at 70 mg/m^2^ free base, and in three patients who initiated at 50 mg/m^2^ free base (Fig. 2). A dose increase was recorded in a further three patients who initiated liposomal irinotecan at 50 mg/m^2^ free base; of these, two patients had subsequent dose reductions (Fig. 2). All patients experienced TEAEs, with 28 patients (96.6%) experiencing TEAEs that were considered treatment related (Table 3). The most frequently reported TEAEs (reported in ≥ 25% of all patients) of any grade were diarrhea (89.7%), fatigue (62.1%), nausea (55.2%), vomiting (41.4%), hypokalemia (37.9%), and decreased appetite (31.0%). Serious TEAEs were reported in 17 patients (58.6%) overall and were considered treatment related in six patients (20.7%). Serious TEAEs reported in at least 10% of all patients were diarrhea (17.2%), nausea (10.3%), and asthenia (10.3%). In total, 21 patients (72.4%) experienced a TEAE with an NCI CTCAE grade of at least 3, with 12 patients (41.4%) experiencing a grade 3 or higher TEAE that was considered treatment related. TEAEs with an NCI CTCAE grade of at least 3 reported in at least 10% of all patients were diarrhea (27.6%), nausea (17.2%), fatigue (13.8%), asthenia (10.3%), and hypokalemia (10.3%); all grade 3. No grade 5 TEAEs were reported; no TEAEs resulted in death. One patient (3.4%) discontinued liposomal irinotecan monotherapy as a result of TEAEs (diarrhea, nausea, and vomiting) that were considered treatment related.

**Table 3:** Patient safety analysis (liposomal irinotecan safety population, *N* = 29)

	Cohort 1 (*n* = 10)	Cohort 2^a^ (*n* = 9)	Cohort 3 (*n* = 10)	Total population (*N* = 29)
Exposure, median (range)				
Treatment duration, weeks	6.1 (0.1–78.4)	12.3 (0.1–38.9)	13.9 (2.1–105.3)	12.3 (0.1–105.3)
AEs, *n* (%)				
Any TEAE	10 (100.0)	9 (100.0)	10 (100.0)	29 (100.0)
TEAEs related to liposomal irinotecan	10 (100.0)	8 (88.9)	10 (100.0)	28 (96.6)
Serious TEAEs	6 (60.0)	4 (44.4)	7 (70.0)	17 (58.6)
Serious TEAEs related to liposomal irinotecan	4 (40.0)	1 (11.1)	1 (10.0)	6 (20.7)
TEAEs with NCI CTCAE grade ≥ 3	8 (80.0)	6 (66.7)	7 (70.0)	21 (72.4)
TEAEs with NCI CTCAE grade ≥ 3 related to liposomal irinotecan	6 (60.0)	3 (33.3)	3 (30.0)	12 (41.4)
TEAEs leading to liposomal irinotecan discontinuation	1 (10.0)	0	0	1 (3.4)
TEAEs leading to liposomal irinotecan discontinuation related to liposomal irinotecan	1 (10.0)	0	0	1 (3.4)
TEAEs leading to death	0	0	0	0
TEAEs leading to death related to liposomal irinotecan	0	0	0	0
TEAEs leading to dose adjustment	7 (70.0)	7 (77.8)	6 (60.0)	20 (69.0)
TEAEs leading to dose adjustment related to liposomal irinotecan	7 (70.0)	4 (44.4)	4 (40.0)	15 (51.7)
Commonly reported TEAEs (> 25% in any cohort)				
Diarrhea	10 (100.0)	9 (100.0)	7 (70.0)	26 (89.7)
Fatigue	6 (60.0)	5 (55.6)	7 (70.0)	18 (62.1)
Nausea	8 (80.0)	5 (55.6)	3 (30.0)	16 (55.2)
Vomiting	6 (60.0)	3 (33.3)	3 (30.0)	12 (41.4)
Hypokalemia	4 (40.0)	3 (33.3)	4 (40.0)	11 (37.9)
Decreased appetite	5 (50.0)	2 (22.2)	2 (20.0)	9 (31.0)
Back pain	4 (40.0)	1 (11.1)	2 (20.0)	7 (24.1)
Alanine aminotransferase increased	3 (30.0)	0	4 (40.0)	7 (24.1)
Anemia	3 (30.0)	2 (22.2)	2 (20.0)	7 (24.1)
Abdominal pain	3 (30.0)	3 (33.3)	1 (10.0)	7 (24.1)
Headache	3 (30.0)	1 (11.1)	2 (20.0)	6 (20.7)
Cough	1 (10.0)	2 (22.2)	3 (30.0)	6 (20.7)
Alopecia	4 (40.0)	2 (22.2)	0	6 (20.7)
Hypocalcemia	3 (30.0)	2 (22.2)	1 (10.0)	6 (20.7)
Asthenia	1 (10.0)	1 (11.1)	3 (30.0)	5 (17.2)
Constipation	3 (30.0)	0	2 (20.0)	5 (17.2)
Dyspnea	1 (10.0)	0	4 (40.0)	5 (17.2)

*AE* adverse event, *NCI CTCAE* National Cancer Institute Common Terminology Criteria for Adverse Events, *TEAE* treatment-emergent adverse event

^a^One patient was enrolled but not treated with liposomal irinotecan and is not included in any safety or efficacy assessments

Based on laboratory abnormalities with an NCI CTCAE grade of 3 or 4, four patients had increased bilirubin, three had hypoglycemia, three had hypokalemia, three had hypophosphatemia, two had low hemoglobin, one had increased aspartate aminotransferase, one had leucopenia, and one had neutropenia (recorded as the MedDRA preferred term, neutrophil count decreased). Based on NCI CTCAE reports of grade 2 neutrophil count decreased, four additional patients had neutropenia, and all five reports of neutropenia were considered treatment related. Two further patients (6.9%) had an actual NCI CTCAE report of grade 2 neutropenia, one of which was considered treatment related.

### Metastatic tumor receptor status—exploratory, post hoc analyses

Among patients who received liposomal irinotecan and had evaluable on-study biopsies, metastatic tumor receptor status was discordant with cohort assignment for four of ten patients in cohort 1 (ER− and PR−, one patient [#8]; HER2+, three patients [#3, #5, #9]) and two of seven patients in cohort 2 (HER2+, one patient [#16]; ER+ and HER2+, one patient [#12]) (Supplementary Table S3).

## Discussion

This study is the first to evaluate the efficacy and safety of liposomal irinotecan specifically in heavily pretreated adult patients with mBC, including those with active BM. Findings from the expansion phase of this open-label, phase I study demonstrate the antitumor activity of liposomal irinotecan monotherapy. Liposomal irinotecan was relatively well tolerated, with only one patient discontinuing because of TEAEs (diarrhea, nausea, and vomiting).

Despite recent improvements in survival rates, mBC remains an incurable disease, and new treatment options are needed, particularly because response rates in late lines of treatment and in patients with BM remain low. Typically, clinical trials of new agents exclude patients with active or untreated BM, and therefore CNS disease remains particularly challenging to treat. However, based on positive findings from the HER2CLIMB trial in patients with HER2+ mBC, which did include patients with BM [16], the HER2 inhibitor tucatinib, in combination with capecitabine and trastuzumab, recently received FDA-approval for the treatment of patients with advanced unresectable or metastatic HER2+ breast cancer, including patients with BM [4]. Neratinib and lapatinib are also small-molecule inhibitors of HER2 [17, 18], and have shown modest activity as single agents or in combination with capecitabine for treating HER2+ mBCBM. The present study evaluated heavily pretreated patients with mBC, including those with active BM; some patients have been living with mBC for more than 5 years. The study population is therefore representative of patients with high unmet need and for whom additional standard treatment options would not be expected to provide robust responses or durable benefit.

Endocrine, targeted, and cytotoxic systemic treatments used as monotherapy in pretreated patients with mBC have been reportedly associated with ORRs ranging from 14 to 32%; combination treatments are associated with improved ORRs [19]. Two small, single-arm clinical trials have specifically assessed the use of non-liposomal irinotecan monotherapy in patients with mBC, with reported ORRs of 5.6% and 23% [20, 21]. Studies of non-liposomal irinotecan in combination with a chemotherapeutic agent in patients with mBC have reported ORRs ranging from 11 to 58.3% [22–29]. In addition, in a small-scale pilot study that assessed the use of multi-omic profiling of target tumors to guide treatment selection in patients with mBC, the most frequently selected treatment was irinotecan based on identified topoisomerase I expression in 12 of 25 evaluated patients (7 received irinotecan combination therapy; 5 received irinotecan monotherapy) [30]. Of these 25 patients, 14 (56%) exhibited clinical benefit (defined as growth modulation index ≥ 1.3) [30]. One phase II study that assessed etirinotecan pegol, a long-acting formulation of irinotecan, reported an ORR of 29% [31]; however, the drug failed to demonstrate superiority to the physician’s choice (single-drug treatment) for overall survival in the randomized phase III BEACON trial [32].

In the present study, the ORR observed with liposomal irinotecan (34.5%) was numerically higher than that historically reported with non-liposomal irinotecan monotherapy [20, 21]. This may be due to the prolonged plasma circulation and EPR effect observed with near-nanoscale liposomal irinotecan, both of which allow for improved tumor drug delivery. Nanoparticle deposition within patients’ intracranial tumor lesions has previously been reported for ferumoxytol as assessed by MRI [15] and liposomes with comparable dimensions and lipid compositions to liposomal irinotecan as assessed by positron emission tomography [33]. The ability of liposomal irinotecan to penetrate the blood-tumor-barrier, in part owing to the small diameter of the liposomes (110 nm), and accumulate in CNS lesions has been demonstrated in non-clinical models of intracranial metastasis in breast cancer [13] and orthotopic glioblastoma models [8, 34]. Liposomes crossed the blood-tumor-barrier and accumulated in brain metastases, but not in normal brain tissue. Extended and preferential accumulation of irinotecan and the active metabolite, SN-38, were observed in these models compared to treatment with non-liposomal irinotecan [8, 13].

In the pilot phase of this study, total SN-38 levels and the ratio of total SN-38:total irinotecan were reportedly sixfold and eightfold higher in tumors than in plasma [12]. In the phase III NAPOLI-1 study in patients with mPDAC, average concentration and duration above threshold concentration for unencapsulated SN-38 in a population pharmacokinetics model were positively correlated with overall response rate, PFS and overall survival in patients receiving liposomal irinotecan with 5-fluorouracil/leucovorin [12]. Thus, the favorable pharmacokinetic characteristics of liposomal irinotecan likely contributed to the efficacy observed in the present study. The ORR with liposomal irinotecan was 30% for CNS disease in cohort 3 (mBCBM), and 50% of patients demonstrated clinical benefit. Two previous open-label, phase I studies that assessed liposomal irinotecan in patients with advanced solid tumors refractory to standard systemic chemotherapy included a small subset of patients with mBC [35, 36]. The observed BOR with liposomal irinotecan was PR in one of four patients with mBC in one study [35], and SD in one of two patients with mBC in the second study [36]. The present study provides additional evidence of the antitumor activity of liposomal irinotecan in a heavily pretreated population with mBC, including BM, supporting further investigation.

ORRs observed in this study were similar across all cohorts (ER+ and/or PgR+/HER2−, TNBC or mBCBM), demonstrating liposomal irinotecan activity in all subtypes of mBC. Notably, of the five patients in cohort 3 who had a PR (non-CNS PR, two patients; CNS PR, two patients; both non-CNS and CNS PR, one patient), three had TNBC (a total of four patients had TNBC in cohort 3), one patient had ER+ or PgR+/HER2+ mBC, and one patient had ER+ or PgR+/HER2− mBC. In these two patients with hormone-receptor-positive mBC with a PR, durable responses were noted for both CNS and non-CNS disease; lasting 40 weeks in the patient with HER2+ mBC, and 104 weeks in the patient with HER2− mBC. These findings warrant further investigation of liposomal irinotecan in patients with mBC and BM, including those with TNBC for whom existing treatment options are extremely limited.

Our exploratory post hoc analyses of metastatic tumor receptor status were consistent with previous reports of the potential for discordance and receptor conversion between primary and metastatic tumors [37], highlighting the need to consider inter- and intra-tumor heterogeneity when selecting treatments [38].

The observed safety profile of liposomal irinotecan was consistent with that reported in earlier studies of non-liposomal and liposomal irinotecan, with gastrointestinal TEAEs, including diarrhea, nausea, and vomiting, being among the most commonly reported grade 3 or 4 TEAEs [20, 21, 24, 25, 28, 35, 36, 39, 40]; no new or unexpected TEAEs were reported. Perhaps the most noticeable difference between previous studies and the present findings is the absence of grade 3 or 4 neutropenia; however, it should be noted that neutropenia was observed in seven patients (NCI CTCAE records of grade 2 neutropenia in three patients, grade 2 neutrophil count decreased in three patients, and grade 3 neutrophil count decreased in one patient). This finding supports the suggestion that liposomal irinotecan monotherapy is associated with better tolerability than liposomal irinotecan combination therapy [41]. In the phase III NAPOLI-1 study, neutropenia was reported in 23.08% of patients receiving liposomal irinotecan 70 mg/m^2^ free base every 2 weeks in combination with 5-fluorouracil/leucovorin [41]. The relatively low frequency of neutropenia observed in the current study may, in part, be due to the dose of liposomal irinotecan and/or the increased localization of liposomal irinotecan within target lesions (rather than in plasma) via the EPR effect [11, 12]. Improved tumor drug delivery via innovative therapeutic platforms, such as nanotherapeutics or antibody drug conjugates (ADCs), are major areas of research for increasing the efficacy of cytotoxic agents while minimizing toxicity. Sacituzumab govitecan-hziy is an ADC comprised of a Trop-2 monoclonal antibody linked to a SN-38 payload, that was recently approved for patients with metastatic TNBC who have received at least two prior lines of therapy for metastatic disease [42, 43]. This agent demonstrated an ORR of 33.3% in a population with a median of three prior lines of treatment, similar to the patient population in the present study [42]. However, this study excluded patients with active, symptomatic, or untreated BM. Thus, the CNS activity of sacituzumab govitecan-hziy is unknown at present. Future research will hopefully continue to elucidate optimal drug delivery mechanisms to maximize the therapeutic potential of irinotecan in mBC, particularly in patients with untreated BCBM.

The main strength of the present study is that liposomal irinotecan was assessed specifically in patients with mBC, including those with active BM. Our findings provide a springboard for further investigation of liposomal irinotecan in this population, in whom alternative therapies are still needed. Study limitations include a small sample size, the open-label study design, and the lack of a comparator group; however, this study showed encouraging antitumor activity in this heavily pretreated population who had experienced disease progression despite multiple lines of chemotherapy.

## Conclusions

Among heavily pretreated patients with mBC with or without BM, liposomal irinotecan monotherapy every 2 weeks had a safety profile consistent with that previously reported for liposomal irinotecan in patients with solid tumors and was associated with an ORR of at least 30%. In future trials, proactive management of gastrointestinal toxicities, such as diarrhea, could improve the risk–benefit profile of liposomal irinotecan. These results suggest that further clinical assessment of liposomal irinotecan is warranted in mBC and active BM.

## Electronic supplementary material

Below is the link to the electronic supplementary material.

Supplementary file1 (DOCX 200 kb)

## Data Availability

If patient data can be anonymized, Ipsen will share all individual patient data that underlie the results reported in this article with qualified researchers who provide a valid research question. Study documents, such as the clinical study report, are not always available. Proposals should be submitted to DataSharing@Ipsen.com and will be assessed by a scientific review board. Data are available beginning 6 months, and ending 5 years, after publication; after this time, only raw data may be available.
