# Supplementary material for: Phase I study of liposomal irinotecan in patients with metastatic breast cancer: findings from the expansion phase
Source: Breast Cancer Res Treat. 2020 Nov 17;185(3):759–71. doi: 10.1007/s10549-020-05995-7 (PMC7921078; doi:10.1007/s10549-020-05995-7)
Supplement: Supplementary file 1 — Supplementary file1 (DOCX 200 kb) [file 10549_2020_5995_MOESM1_ESM.docx]

# Supplementary materials

## Methods

### Ethics

The study protocol and amendments, patient information leaflet, and informed consent form and updates thereof were reviewed and approved by an Institutional Review Board, prior to commencement of the study and during the study, when applicable. The study was conducted under provisions of the Declaration of Helsinki in accordance with the International Conference on Harmonisation Consolidated Guidance on Good Clinical Practice and in compliance with independent ethics committees/Institutional Review Boards and informed consent regulations.

### Expansion phase treatment

Patients were premedicated with standard doses of dexamethasone and a 5-HT3 antagonist or other antiemetics according to institutional standard practices for irinotecan administration. Throughout the expansion phase, atropine could be prescribed prophylactically for patients who experienced acute cholinergic symptoms with previous liposomal irinotecan infusions.

### Expansion phase efficacy assessments

Additional efficacy outcomes were derived based on the tumor response assessments. Best overall response (BOR): defined as the best response (Response Evaluation Criteria in Solid Tumors [RECIST] v1.1 or modified RECIST [mRECIST]) recorded from first dose to disease progression or start of new anticancer therapy and/or surgery. Objective response rate: defined as the proportion of patients with a BOR, characterized as either a complete or partial response, relative to the total number of evaluable patients. Clinical benefit rate: defined as the proportion of patients with a BOR, characterized as either a complete or partial response at any time, or stable disease for at least 24 weeks, relative to the total number of evaluable patients. Duration of objective response: defined as the time from first documentation of response (complete or partial response) to the date of disease progression or death due to any cause – whichever occurred first. Progression-free survival: defined as the time from first dose of liposomal irinotecan to the date of radiological disease progression (RECIST v1.1 or mRECIST) or death due to any cause.

### Metastatic tumor receptor status – exploratory analysis

Estrogen receptor (ER)/progesterone receptor (PgR) status and human epidermal growth factor receptor 2 status of on-study biopsies (see supplementary materials for Ramanathan et al. 2017 [14]) of metastatic tumors from cohorts 1 and 2 were evaluated retrospectively by the Food and Drug Administration-approved Dako ER/PgR pharmDx-IVD kit and the Dako HercepTest® IVD kit by Flagship Biosciences Inc (Westminster, CO, USA), CAP/CLIA laboratory, as per the American Society of Clinical Oncology/College of American Pathologists testing guidelines. Where available from multiple passes from a single lesion, stained tumor biopsies from 19 patients in cohorts 1 and 2 were evaluated by a reviewing-board-certified MD anatomical pathologist and scored according to the manufacturer’s instructions. Receptor status was compared between on-study biopsies and that recorded from archived lesion tissue samples as well as re-evaluation of archival samples where available.

Supplementary Fig. 1 CONSORT DIAGRAM – patient disposition flowchart for the expansion phase


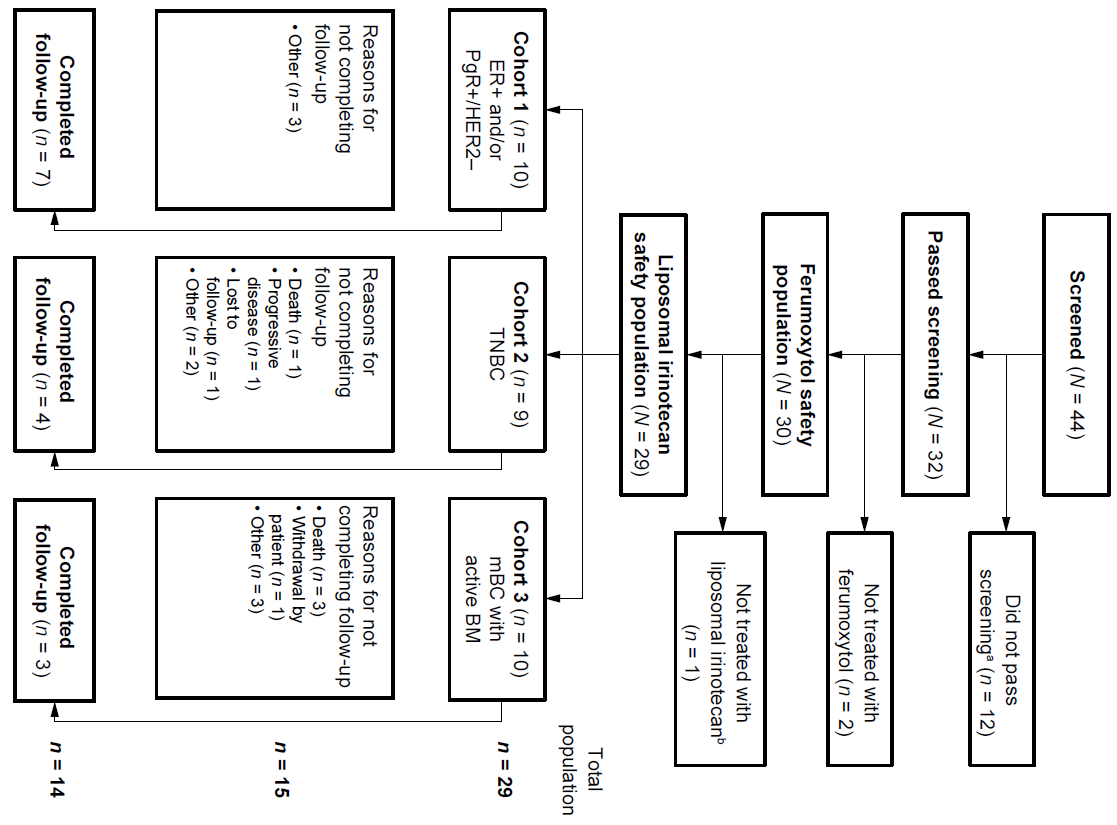


^a^Data from the patients who did not pass screening were entered into the clinical database but were not presented in the final patient data listings

^b^One patient died owing to progressive disease before initiating treatment with liposomal irinotecan

*BM* brain metastases, *ER* estrogen receptor, *HER2* human epidermal growth factor receptor 2, *mBC* metastatic breast cancer, *PgR* progesterone receptor, *TNBC* triple-negative breast cancer

Supplementary Table S1 Inclusion and exclusion criteria

|  | Inclusion criteria | Exclusion criteria |
| --- | --- | --- |
| All patients | - Pathologically confirmed diagnosis of solid tumors - Documented locally advanced or metastatic disease with at least two radiologically measurable lesions - Eastern Cooperative Oncology Group performance status of 0–1 - Adequate bone marrow, hepatic, and renal function - Normal electrocardiogram - Aged ≥ 18 years - Received ≥ 1 cytotoxic therapy in the locally advanced and metastatic setting, with exception of TNBC patients who had progressed within 12 months of adjuvant therapy^a^ - Received ≤ 5 prior lines of chemotherapy in the metastatic setting (no limit to prior lines of hormonal therapies in ER+/PgR+ tumors)^a^ - Candidate for chemotherapy^a^ | - Clinically significant gastrointestinal disorders - Prior irinotecan or bevacizumab therapy in the past 6 months - Prior treatment with any topoisomerase I inhibitor (irinotecan derived or topotecan)^a^ - History of any second malignancy in the past 3 years - Known hypersensitivity to irinotecan or ferumoxytol - Inability to undergo MRI - Concurrent illnesses that were a relative contraindication to study participation, such as active cardiac or liver disease - Active infection or an unexplained fever > 38.5°C - Prior chemotherapy administered in the 3 weeks, or in a time interval ≤ 5 half-lives of the agent (whichever is longer), prior to the first scheduled day of dosing in this study - Received radiation therapy in the past 14 days - Treated with parenteral iron in the past 4 weeks - Patients who are positive for the human immunodeficiency virus and were receiving combination antiretroviral therapy, or those with other conditions requiring treatment with which there was potential for ferumoxytol to have negative pharmacokinetic interactions - Pregnant or breastfeeding |
| Cohort 1 | - HER2− breast cancer with ER+ and/or PgR+ tumors^a^ - ≥ 1 lesion amenable to multiple-pass core biopsies^a^ | - Active central nervous system metastasis, indicated by clinical symptoms, cerebral edema, or steroid requirement (applicable to cohorts 1 and 2) |
| Cohort 2 | - HER2− breast cancer with ER− and PgR− tumors (TNBC)^a^ - ≥ 1 lesion amenable to multiple-pass core biopsies^a^ |  |
| Cohort 3 | - Any subtype of mBC with active BM^a^ - Radiographic evidence of new or progressive BM after radiation therapy with ≥ 1 lesion measuring ≥ 1 cm in the longest dimension on gadolinium-enhanced MRI (note: progressive brain lesions are not required to meet RECIST criteria in order to be eligible; extra-cranial metastatic disease is also allowed)^a^ - Imaging following prior radiation is not consistent with pseudo-progression in the judgment of the treating clinician^a^ - Neurologically stable as defined by^a^:   - stable or decreasing dose of steroids and anti-convulsants for ≥ 7 days prior to study entry   - no clinically significant mass effect, hemorrhage, midline shift, or impending herniation on baseline brain imaging   - no significant focal neurologic signs and/or symptoms which would necessitate radiation therapy or surgical decompression in the judgment of the treating clinician - No evidence of diffuse leptomeningeal disease on brain MRI or by previously documented cerebrospinal fluid cytology (note: discrete dural metastases are permitted)^a^ | – |

^a^Inclusion/exclusion criterion specific to the expansion phase population.

*BM* brain metastases, *ER* estrogen receptor, *HER2* human epidermal growth factor 2, *mBC* metastatic breast cancer, *MRI* magnetic resonance imaging, *PgR* progesterone receptor, *RECIST* Response Evaluation Criteria in Solid Tumors, *TNBC* triple-negative breast cancer

Supplementary Table S2 CNS response assessment using modified RECIST criteria [7]

| Response | Definition |
| --- | --- |
| Complete response | Disappearance of all target and non-target lesions; or lesions do not show any gadolinium enhancement and are completely necrotic |
| Partial response | ≥ 30% decrease in the sum longest dimension of target lesions (taking the baseline sum diameters as reference) *and* an absolute decrease of ≥ 5 mm in ≥ 1 target lesion; non-target lesions do not meet the criteria for progressive disease |
| Stable disease | Neither sufficient shrinkage to qualify for partial response nor sufficient increase to qualify for progressive disease, taking the smallest sum diameters while on study as reference; non-target lesions do not meet the criteria for progressive disease |
| Progressive disease | ≥ 20% increase in the sum longest dimension of target lesions (taking the smallest sum while on study as reference, including the baseline sum if that is the smallest on study) *and* an absolute increase in size of ≥ 5 mm in ≥ 1 target lesion, *or* the appearance of ≥ 1 new lesion of ≥ 6 mm in size; and/or growth of non-target lesion(s) sufficient to determine unequivocal progression |

*CNS* central nervous system, *RECIST* Response Evaluation Criteria in Solid Tumors

Supplementary Table S3 Metastatic tumor expression of HER2, ER, and PgR in cohorts 1 and 2

| Study cohort | Patient | Responder (complete or partial response) | ER summation score/status | PgR summation score/status | Binned HER2 score/status^a^ | Metastatic tumor receptor status concordant with assigned cohort^b^ |
| --- | --- | --- | --- | --- | --- | --- |
| Cohort 1  (ER+ and/or PgR+/ HER2−) | 1 | Yes | 8/Positive | 5/Positive | 1+/Negative | Yes |
|  | 2 | Yes | 6/Positive | 0/Negative | 0/Negative | Yes |
|  | 3 | Yes | 4/Positive | 0/Negative | 1+/Negative | Yes (A) |
|  |  |  | 8/Positive | 0/Negative | 3+/Positive | No |
|  | 4 | Yes | 8/Positive | 6/Positive | 1+/Negative | Yes |
|  |  |  | 8/Positive | 7/Positive | 1+/Negative | Yes |
|  | 5 | No | 8/Positive | 5/Positive | 3+/Positive | No |
|  | 6 | No | 8/Positive | 5/Positive | 2+/Negative | Yes |
|  | 7 | No | 8/Positive | 7/Positive | 2+/Negative | Yes |
|  | 8 | No | 0/Negative | 0/Negative | 0/Negative | No |
|  |  |  | 0/Negative | 0/Negative | 0/Negative | No |
|  | 9 | No | 8/Positive | 4/Positive | 3+/Positive | No |
|  | 10 | No | 7/Positive | 0/Negative | 1+/Negative | Yes |
| Cohort 2  (TNBC) | 11 | Yes | 0/Negative | 0/Negative | 1+/Negative | Yes |
|  |  |  | 0/Negative | 0/Negative | 1+/Negative | Yes |
|  | 12 | Yes | 6/Positive | 0/Negative | 3+/Positive | No |
|  |  |  | 4/Positive | 0/Negative | 2+/Negative | No |
|  | 13 | Yes | 0/Negative | 0/Negative | 2+/Negative | Yes (A) |
|  |  |  | 0/Negative | 0/Negative | 2+/Negative | Yes |
|  | 14 | No | 0/Negative | 0/Negative | 1+/Negative | Yes |
|  | 15 | No | 0/Negative | 0/Negative | 0/Negative | Yes (A) |
|  | 16 | No | 0/Negative | 0/Negative | 3+/Positive | No |
|  | 17 | No | 0/Negative | 0/Negative | 0/Negative | Yes |
|  |  |  | 0/Negative | 4/Positive | 0/Negative | No (A) |
|  | 18 | No | 0/Negative | 0/Negative | 0/Negative | Yes |
|  |  |  | 0/Negative | 0/Negative | 1+/Negative | Yes |
|  | 19 | *No evaluable biopsies available* | | | | |
|  | N/A (this patient was enrolled in the study and received ferumoxytol, but died before receiving liposomal irinotecan) | NA | 3/Positive | 0/Negative | NA^c^ | NA |
|  |  |  | 3/Positive | 0/Negative | 0/Negative | No |
|  |  |  | 3/Positive | 0/Negative | 0/Negative | No |

(A), indicates archival tissue sample.

^a^As per the study protocol, a binned HER2 score of 0, 1+, or 2+ was considered to be negative

^b^Concordance in receptor status was assessed between historical archival receptor subtyping used for cohort assignment and on-study biopsy materials as well as archival material, where available

^c^Tumor samples could not be assessed

No evaluable on-study biopsies were available for patients 15 and 19 from cohort 2. Stained on-study or archival tumor biopsy materials were evaluated by a reviewing-board-certified MD anatomical pathologist. HER2 status was assessed using the HercepTest, and stained tissues were assigned a binned HER2 score ranging from 0 (negative) to 3+ (positive). ER/PgR status was assessed using the ER/PgR pharmDX, and stained tissues were assigned a summation score (0, 2: negative; ≥ 3: positive). The summation score was generated by adding together the proportion score (range: from 0 [negative; no stained cells] to 5 [positive; > 2/3 to 1/1 stained cells]) and intensity score (range: from 0 [no staining] to 3 [strong staining]). All samples were scored according to the manufacturer’s instructions

*ER* estrogen receptor, *HER2* human epidermal growth factor 2, *NA* not applicable, *PgR* progesterone receptor, *TNBC* triple-negative breast cancer
